# Patient facing decision support system for interpretation of laboratory test results

**DOI:** 10.1186/s12911-018-0648-0

**Published:** 2018-07-20

**Authors:** Georgy Kopanitsa, Ilia Semenov

**Affiliations:** 1 Institute Cybernetic Center, Tomsk Polytechnic University, Lenina 30, 634050 Tomsk, Russia; 2 Tomsk State University for Architecture and Building, Tomsk, Russia; 3 Medlinx LLC, Saint-Petersburg, Russia

**Keywords:** Decision support, Laboratory information system, Telemedicine, First order predicates, User acceptance

## Abstract

**Background:**

In some healthcare systems, it is common that patients address laboratory test centers directly without a physician’s recommendation. This practice is widely spread in Russia with about 28% of patients who visiting laboratory test centers for diagnostics. This causes an issue when patients get no help from the physician in understanding the results.

Computer decision support systems proved to efficiently solve a resource consuming task of interpretation of the test results. So, a decision support system can be implemented to rise motivation and empower the patients who visit a laboratory service without a doctor’s referral.

**Methods:**

We have developed a clinical decision support system for patients that solves a classification task and finds a set of diagnoses for the provided laboratory tests results.

The Wilson and Lankton’s assessment model was applied to measure patients’ acceptance of the solution.

**Results:**

A first order predicates-based decision support system has been implemented to analyze laboratory test results and deliver reports in natural language to patients. The evaluation of the system showed a high acceptance of the decision support system and of the reports that it generates.

**Conclusions:**

Detailed notification of the laboratory service patients with elements of the decision support is significant for the laboratory data management, and for patients’ empowerment and safety.

**Electronic supplementary material:**

The online version of this article (10.1186/s12911-018-0648-0) contains supplementary material, which is available to authorized users.

## Background

In some healthcare systems, it is common that patients address laboratory test centers directly without a physician’s recommendation [1]. This practice is widely spread in Russia with about 28% of patients who visiting laboratory test centers for diagnostics [2]. This causes an issue when patients get no help from the physician in understanding the results. Patients face a problem when they need to decide how to continue the diagnostics and treatment process. A possible solution to this problem could be that a laboratory test center not only delivers the test results but also their explanation to the patients. This, however, should be done automatically, or at least semi-automatically, to exclude a critical load on the test centers. Clinical decision support systems can become a good technology for an automatic interpretation of test results [3, 4]. The experience in implementation of decision support systems for health care professionals shows their efficiency for medical diagnostics. However, patients require a different approach in data presentation and interpretation [5–11].

Studies [12–15] have demonstrated that many providers do not have systems that can ensure that the test results are reliably communicated to patients. As shown in [16, 17] normal and abnormal test results are commonly missed, even when a health care system widely uses electronic health records (EHRs), and providers miss 1–10% of abnormal test results. It would not be an exaggeration to say that we do not have sufficient mechanisms to ensure that test results are consistently delivered to patients and understood by them.

As a problem importance is recognized, a number of potential solutions has been studied [18–23]. The first approach originates from the development of computerized decision support systems that support test centers in reviewing results and notifying patients in case of abnormal results [18, 19, 21, 23]. Another approach has involved implementation of such testing processes where test centers consistently deliver test results directly to patients. Such systems vary from sending each piece of the test results by mail to complex patient web portals, where they can have access to the history of test results [20, 24].

Interpretation of the test results is a resource consuming task that delays the results and increases costs of each test [25, 26]. However, the computer decision support systems proved to solve such tasks efficiently. To increase motivation and support the patients who refer to a test center without a doctor’s referral in making better informed decisions, a computer decision support system can be designed and implemented.

The goal of this study is to develop and evaluate a decision support system for patients, which:

- provides a personalized tool to inform patients on the results of the laboratory tests
- empowers patients to form opinions on how to continue or not to continue with a treatment
- prepares patients to have an informed discussion with their doctor.

To support patients, we have implemented and evaluated a decision support system that automatically generates interpretations for laboratory test results:

This paper focuses on the evaluation of correctness and user acceptance of a decision support system for patients of a test center in Saint-Petersburg, Russia.

## Methods

### Implementation

We have developed a clinical decision support system for the patients that solves a classification problem by connecting test results to a list of diagnoses. The decision support is based on a classification algorithm, which produces the following conclusions:

- Located a list of diagnoses that can be related to the test results;
- Found no fitting diagnoses;

To enable a definition of inference rules we have developed a knowledge representation language that is based on the predicate calculus [27] and a user interface to allow medical professionals defining the system rules. For the pilot project, we have chosen a limited set of laboratory tests that could be automatically interpreted by the system. We have interviewed 3 laboratory physicians and 3 specialist physicians (gynecologist, urologist and general practitioner) to define the inference rules for the system.

The decision support system has been implemented and operating in the Helix laboratory center in Saint-Petersburg, Russia.

The system has been implemented using the following technologies:

1. User interfaces and a back-end are based on the. NET Core 2.0
2. Data storage is based on PostgreSQL

### Evaluation

#### Accuracy of the decision support

To evaluate accuracy of the results produced by the system, we have performed a validation of 1000 randomly generated reports. The reports were generated in a way to allow validating all of 89 decision support algorithms. The reports were given to two independent pathology experts to be reviewed independently. The results of the expert review were used to calculate the following criteria [28]:

1. Error rate as an average classification error
2. Accuracy as an average effectiveness of a classifier
3. Precision ((All terms – Mistakes)/All terms),
4. Recall (ratio of true positives to (true positives + false negatives)), and
5. F-measure ($$ 2\bullet \frac{recall\bullet precision}{recall+ precision} $$).

The reviewers’ disagreements were settled by consensus. Cohen’s kappa was calculated to rate the disagreement between reviewers [29].

#### User acceptance

To assess the user acceptance of the system, a Wilson and Lankton’s model of patients’ acceptance of electronic health solutions was applied [30]. The model allowed measuring the following criteria: behavioral intention (BI) to use, intrinsic motivation (IM), perceived ease-of-use (PEOU), and perceived usefulness (PU) of the decision support system.

BI represents the intention to utilize the system and to rely on the decision support that it provides; IM represents the willingness to use the system provided that no direct compensation is available; PEOU represents the extent to which the provided reports are clearly presented and comprehended by users; and PU denotes the degree to which the patients believe that the utilization of the decision support system will improve their experience with laboratory tests.

We have applied a Wilson’s and Lankton [30] revision of the Davis’s et al. [31] method to measure BI, PEOU, and PU. Intrinsic motivation was measured by utilizing the Davis’s et al. method [31].

#### Questionnaire

We started with detection of possible items for the questionnaire by collecting a large list of acceptance test questions. The questions were collected from preceding internal studies, from the literature and from brainstorming. The list was then reviewed by the study team to eliminate the items that do not help to reach the goals of the study and duplicate questions. The remaining items were simplified and worded as clear to the potential participants as possible.

BI measure consisted of 2 objects whereas IM, PEOU, and PU consisted of 3 objects each. Russian translation of the questionnaires made by the research team was used during the study. To rate each item a Likert scale from 1 (not at all) to 7 (very much) was applied [32].

#### Recruitment

The recruitment of the study participants was done in Saint-Petersburg, Russia. The patients were eligible to be invited if they had experience using the system with a minimum of 5 reports on the test outcomes. The recruitment was done by sending invitations to the 500 eligible patients. Later, we have formed a group of 120 patients based on the first responded – first included principal with a recruitment rate of 24%.

Demographic characteristics of the study participants are shown in Table 1. We assessed Information technology (IT) literacy of the patients based on how frequently they use smartphones or personal computer. We assessed IT literacy on the scale from beginners – patients who started using computer or smartphone maximum 6 months before the study begin; intermediate – computer or smartphone users who do it at least 2 times a week; and advanced – daily users of a computer or a smartphone.

**Table 1:** Demographic characteristics of the study participants

Gender	Average age	Age > 60	Education			IT habits		
			Higher	Secondary	Below secondary	Beginners	Intermediate	Advanced
56 Males	41.3	16	18	28	10	6	34	16
64 Females	42.3	12	22	36	6	16	8	0
Total 120		28	30	64	16	22	42	16

#### Data collection and analysis

All the study participants were given individual access to the online questionnaire, which they were asked to fill in (please see Additional file 1 for the questionnaire details). All the patients received a written detailed instruction on how to operate with a questionnaire and the sense of the rating scale.

GNU Octave [33] version 4.0.2 was applied to calculate the statistics of the participants’ general characteristics and user acceptance measurements.

#### Ethics commission approval

The study was approved by the ethics commission of the committee of healthcare of Saint-Petersburg, Russia. All the study participants were informed in written form about the goals of the study and about the meaning of the questionnaires. We assured in written form every participant of their rights to anonymity and confidentiality. Written consent was obtained from every participant. Every participant was informed in written form about a right to withdraw personal data from the study record for up to 3 months after their approval.

## Results

### Implementation

**Fig. 1:**
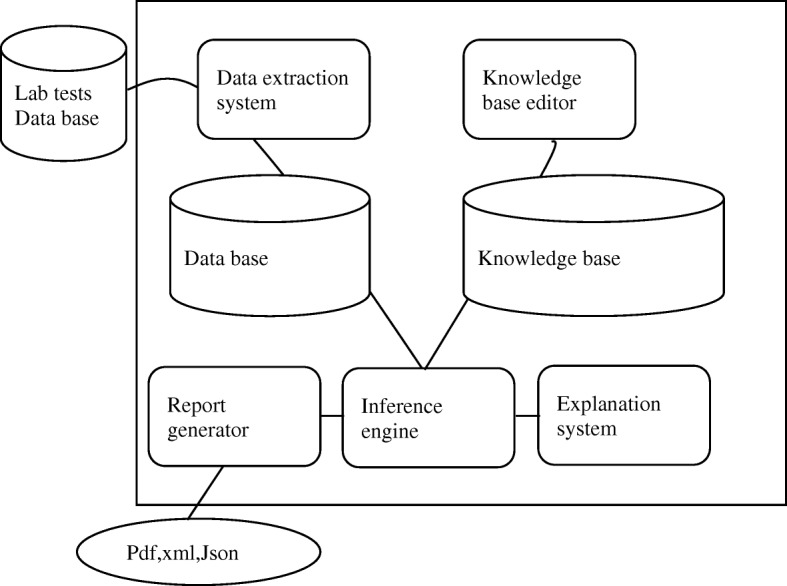
Structural scheme of the decision support system

The clinical decision support system consists of the following modules, which provide the main features of the system (Fig. 1):

- A Data extraction system receives data from external sources, such as hospital or laboratory information systems. It checks the syntax validity of data and sends it to a Database
- A Data base receives and saves facts from an external laboratory information system.
- A Knowledge base editor (see section “User acceptance”.1) provides an interface to experts to define inference rules that are sent to a Knowledge base.
- A Knowledge base stores inference rules.
- Inference engine applies rules from the knowledge base to the facts from the Database to conclude the results and sends them to the explanation system and report generator.
- Explanation system scrutinizes the sequence of the applied inference rules to demonstrate how the result has been achieved.
- Report generator creates a readable report from the inference results and sends it to the report storage.

The example of how the system operates is presented in section “Inference example”.

**Fig. 2:**
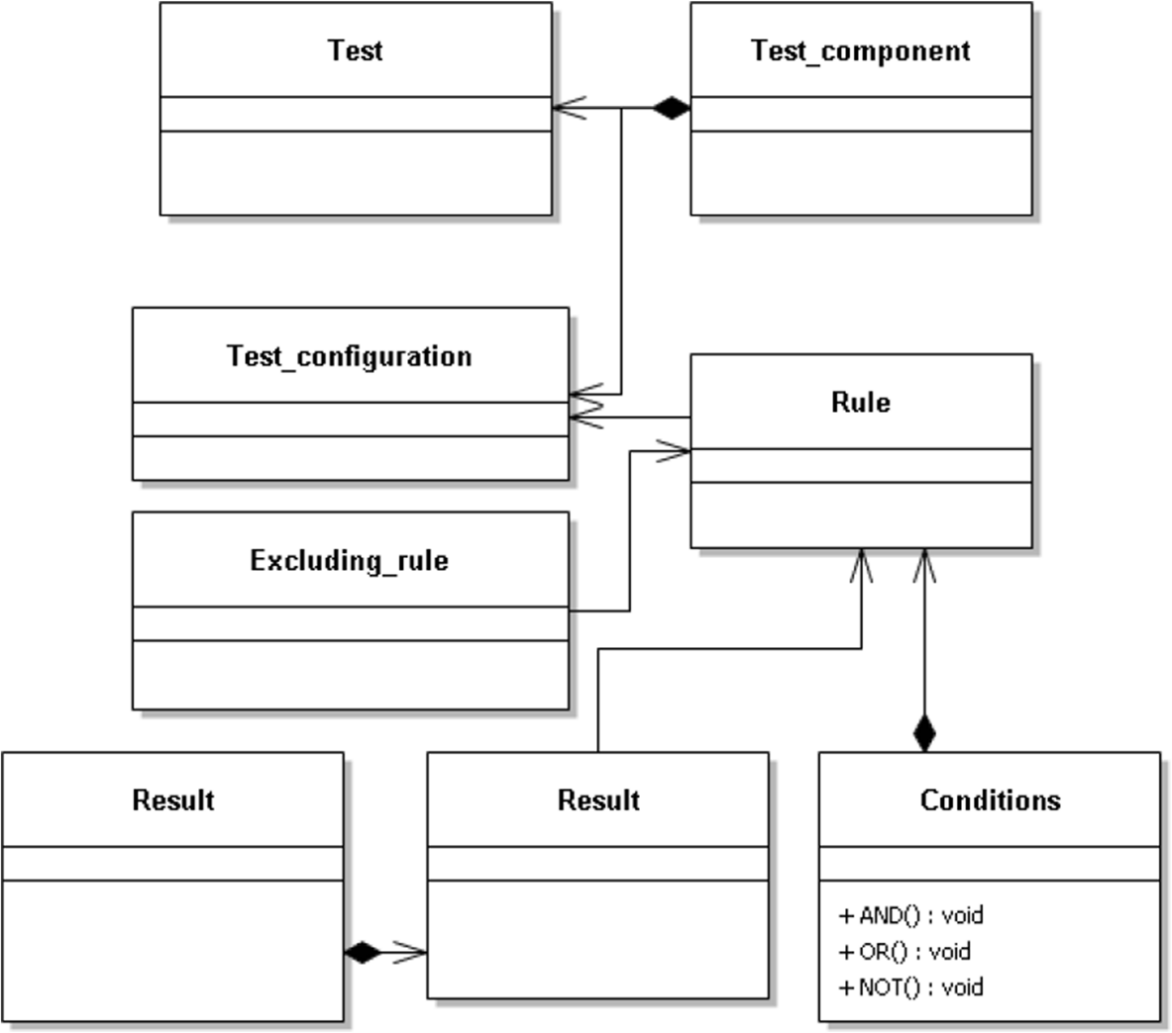
Object model of the system

The knowledge representation language of the system is built upon a first order predicate logic. The scheme of the knowledge base is shown in Fig. 2.

- The main object that the system processes is a laboratory test configuration that consists a laboratory test object and of a list of direct inference rules that can be related to this object.
- Laboratory test object is a model that comprises a list of atomic components of the test e.g. a complete blood count test includes 22 atomic components.
- For each component of a test, we define a list of direct inference rules that have conditions for including this components in the inference. The conditions are represented as comparison operators: =, <>, includes (> = or = <), excludes (> = and = <). Within a rule, the conditions are related by logical operators “and”, “or” and “not”.
- For each direct rule, an expert can model a list of exclusion rules to exclude a direct rule from an inference process if the exclusion conditions are met.
- An order the object groups laboratory tests reflect the commercial orders that patients actually make.

**Fig. 3:**
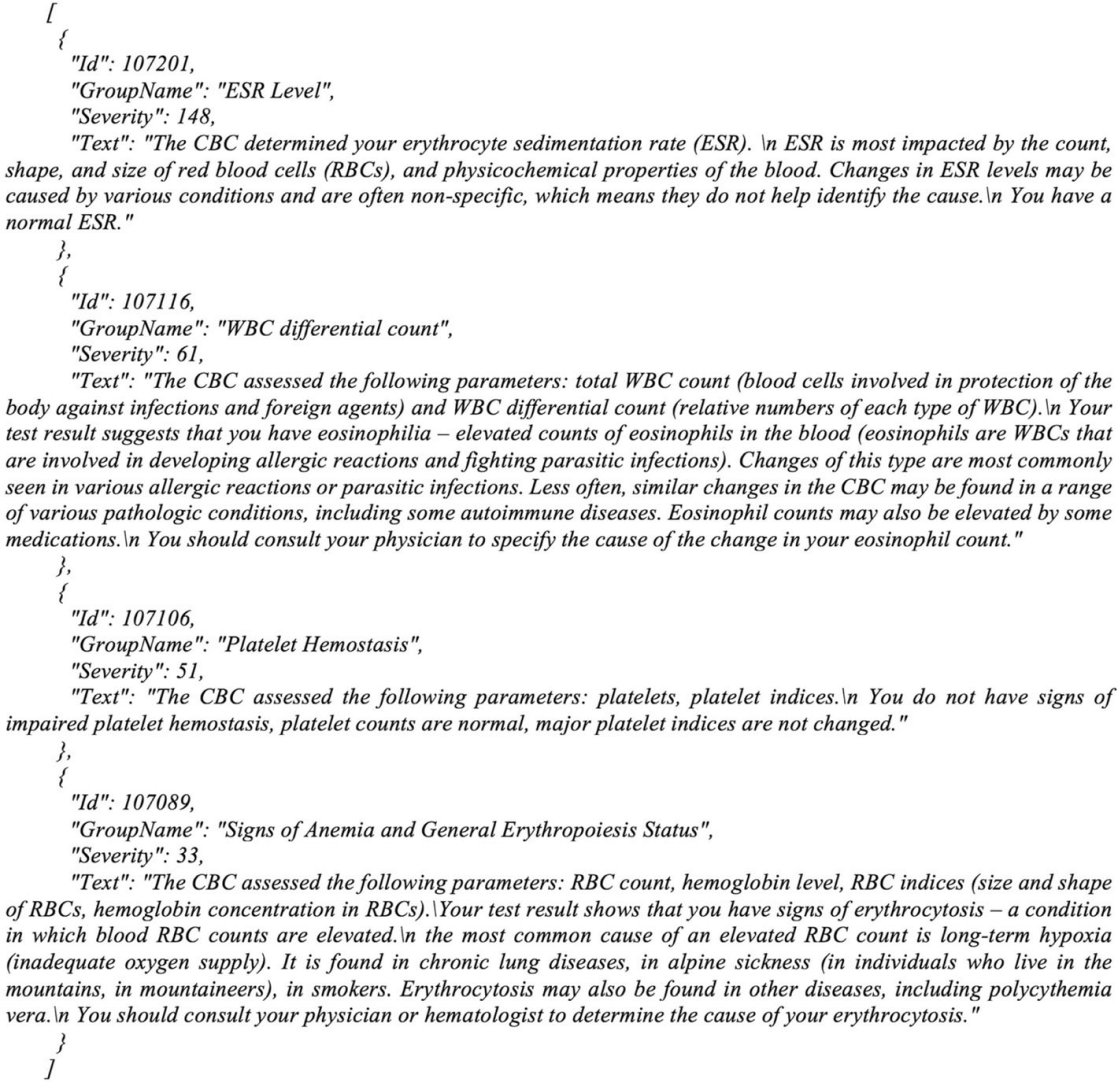
Results of the inference in a json format

General inference process is divided into the following steps:

1. When the system receives an order bundle from a laboratory information system, the tests in the order are being analyzed to generate a list of tests, configurations for which are available in the knowledge base.
2. Actual test results are loaded to the Database of the system and become available for an inference process.
3. The inference engine receives the list of tests and selects proper direct inference rules and exclusion rules in the proper sequence, which can be applied to the received facts.
4. If a direct rule has been successfully applied and no exclusion rule is effective, the inference engine adds a text artefact to the resulting json file (Fig. 3).
5. After the inference has been completed, the resulting json file is sent to the reporting service to generate a pdf report.

The decision support system has been implemented in the Helix laboratory service in Saint-Petersburg, Russia. The system is in commercial production now generating about 20,000 reports a day.

The system has inference algorithms for the following International Statistical Classification of Diseases and Related Health Problems (ICD) 10 groups:

1. Kidney:
  1.1. N30 Cystitis
  1.2. N04 Nephrotic syndrome
  1.3. N39 Other disorders of urinary system
  1.4. N10 Acute pyelonephritis
2. Liver:
  2.1. K75 Other inflammatory liver diseases
  2.2. K72 Hepatic failure, not elsewhere classified
  2.3. K71 Toxic liver disease
  2.4. K81 Cholecystitis
3. Pancreas:
  3.1. K85 Acute pancreatitis
4. Thyroid gland
  4.1. E05 Thyrotoxicosis
  4.2. E03 Other hypothyroidism
5. Red blood cells:
  5.1. D50 Iron deficiency anemia
6. White blood cells:
  6.1. D72 Other disorders of white blood cells
7. Prostate:
  7.1. N41 Inflammatory diseases of prostate

#### Inference example

The full json code of rules and artefacts for the blood sugar testis presented in Additional file 2. The input of the inference is a bundle of resources that has been extracted from a laboratory tests database and were added to the decision support system database (Fig. 1):

1. Patient
2. Observation: Concentration of HbA1C, mmol/mol
3. Observation: Concentration of HbA1C, %
4. Observation: Concentration of Hb, mmol/mol
5. Observation: Concentration of Glucose in Plasma, mmol/L
6. Observation: Concentration of C-Peptide, pmol/L

**Fig. 4:**
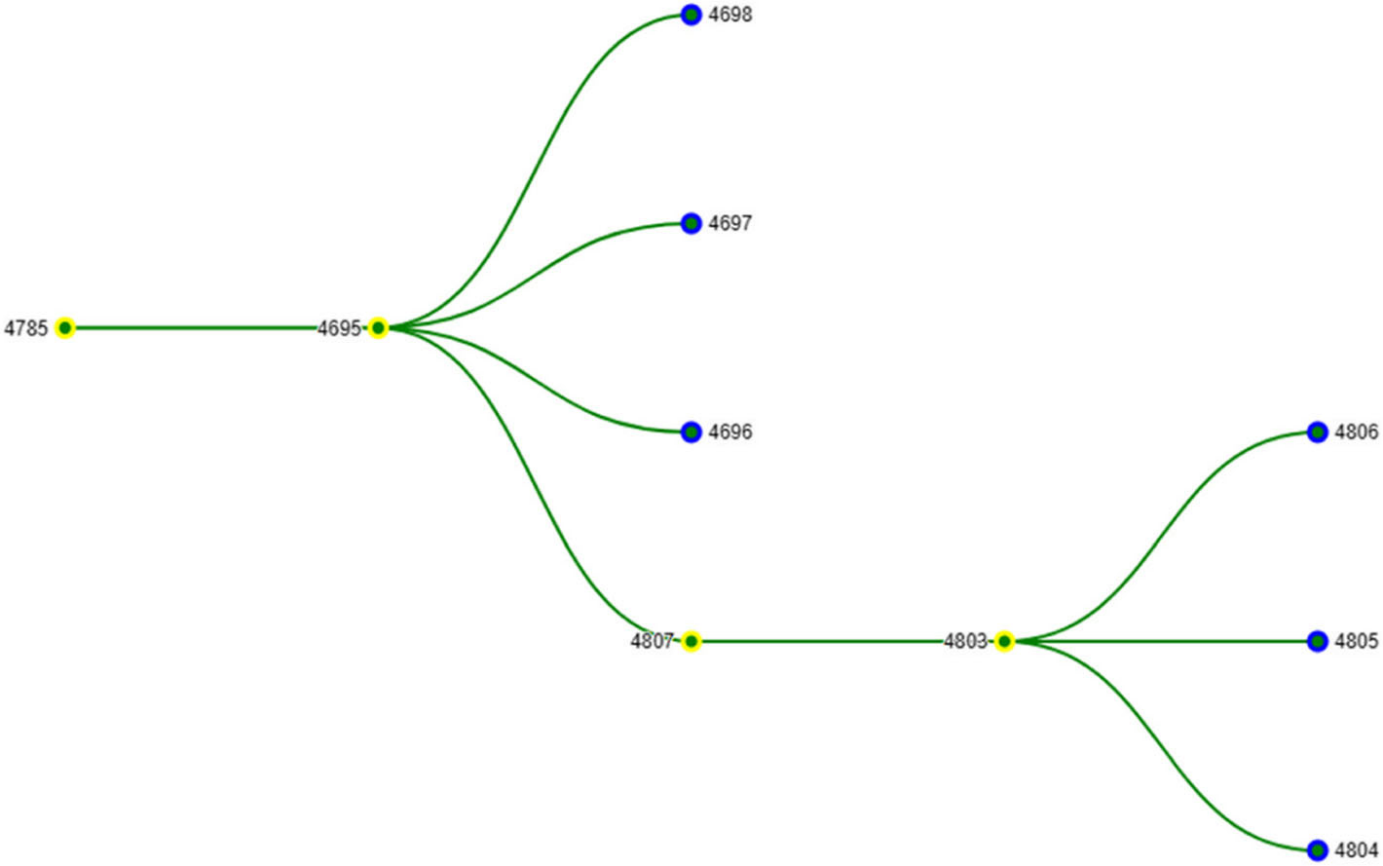
Inference rules sequence

After the system receives the actual values, an inference engine starts building an inference sequence (see Additional file 2. Inference sequence for the rules details and Fig. 4 for the graphical representation of the sequence) based on the available rules from the knowledge base (Fig. 1).

The inference ends up with the conclusion id = 4785 with the artefact id = 4786. The found artefact is added to the generated report, which is then sent to the report generator to create a human readable pdf file. The resulting rules sequence is being visualized by the explanation system (Fig. 1).

#### User interaction

The decision support system consists of 2 main interfaces: for experts to model knowledge and inference rules and for patients to have access to the test results and their interpretation.

#### Expert’s interface

We have developed a web knowledge management application that provides the following features:

- Create and edit inference rules
- Group inference rules
- Create and edit artefacts with doctor’s recommendations that form a decision support report as a result of a logical inference.

**Fig. 5:**
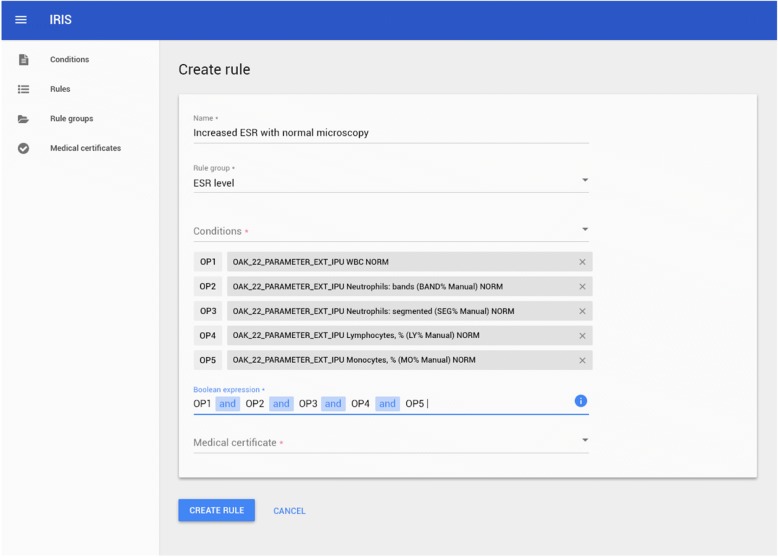
Create rule interface for an expert

**Fig. 6:**
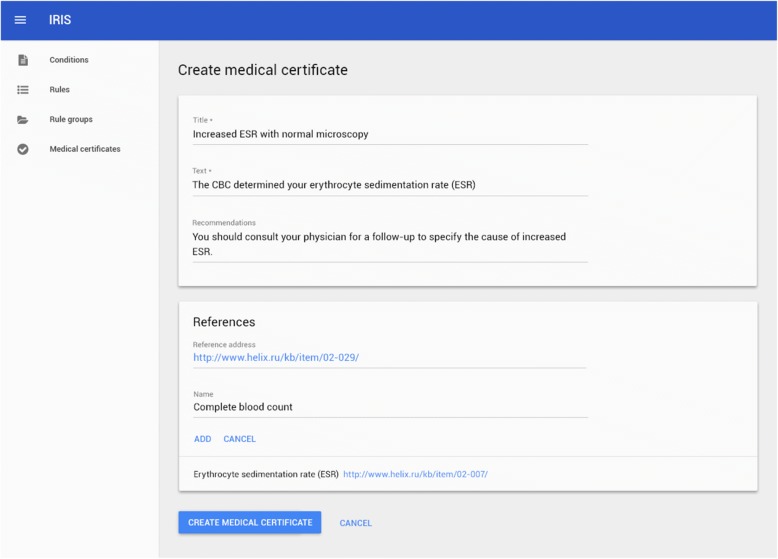
Expert’s interface for recommendations

Fig. 5 shows an inference rule creation screen. A rule consists of several conditions connected by logical operators and a resulting artefact, which represents a text with recommendations to the patient. The artefacts can be created using an interface from Fig. 6.

#### Patient’s user interface

**Fig. 7:**
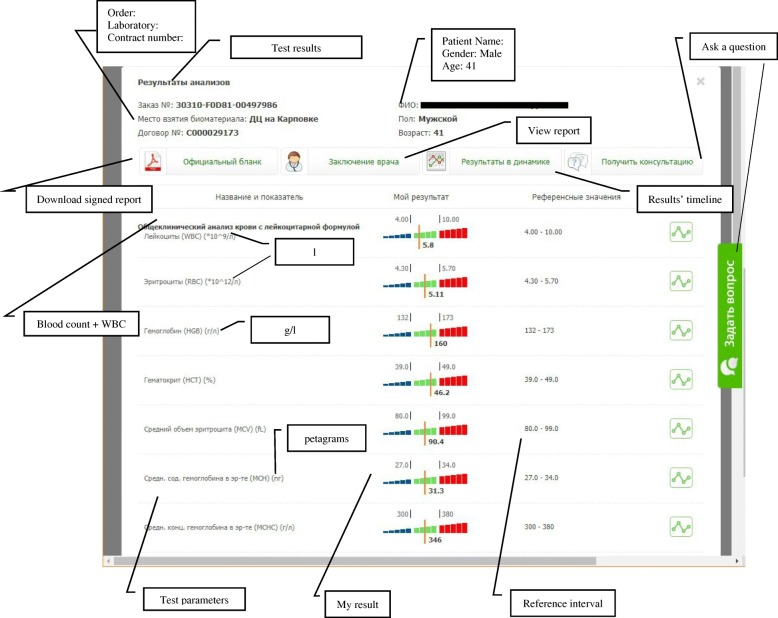
Personal space for a patient on the online portal

**Fig. 8:**
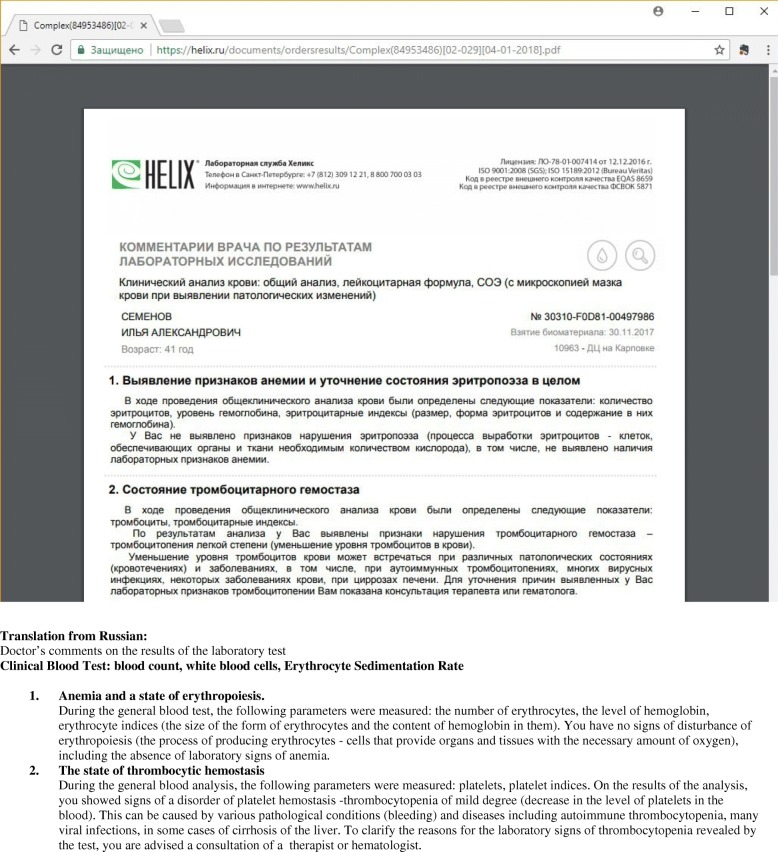
Report, produced by a decision support system

Patient has access to the test results through a web portal, where a list of available tests is provided. For each test, a patient can have an overview of the results (Fig. 7). The results are presented in the table form with the following columns: Parameter name, My Results and a Reference interval. A patient can click on the “Generate report” button (second button from the left with a doctor icon on the Fig. 7) to open a decision support report (Fig. 8).

### Evaluation

#### Correctness

A sample of 1000 reports was independently assessed by two independent pathology experts. The results of assessment for each criterion are shown in Table 2. The experts revealed disagreement in the assessment of 2 reports.

**Table 2:** Reports’ quality evaluation

Analyzed reports	Error rate	Accuracy	Precision	Recall	F-measure	Cohen’s kappa
1000	7 (0.7%)	0.	0.99	0.99	0.99	0.99

#### Acceptance

The mean values for BI, IM, PEOU, and PU (5.9, 6.2, 5.7, and 5.9 respectively) showed a high acceptance of the decision support system and the reports that it generates (Table 3).

**Table 3:** Acceptance criteria

Criterion, Item	Entire group (120 participants)				> 60 (28 participants)				< 60 (92 participants)			
	Mean	Median	Max	Min	Mean	Median	Max	Min	Mean	Median	Max	Min
Behavioral intention to use	5.9				5.6				6.1			
I intend to use the tool to understand my test results	5.7	5	7	4	5.5	5.5	6	4	5.8	6	7	5
I feel like I will use it in the future	6.1	5.5	7	5	5.7	5.5	7	5	6.3	6	7	5
Intrinsic motivation	6.2				6.1				6.2			
I find the system useful for me	6.2	6	7	6	6.0	6	7	6	6.2	6	7	6
The system helps me to make more informed decisions	5.9	5.5	7	4	5.7	5.5	6	4	6.0	6	7	5
The system is reliable and I trust it	6.4	6	7	5	6.3	6	6	5	6.4	6	7	5
Perceived ease of use	5.7				5.6				6.1			
The reports are clear and understandable	6.3	6	7	6	6.2	6	7	6	6.4	6.5	7	6
It is easy to access the reports	5.7	6	7	5	5.5	5.5	7	5	5.8	6	7	6
I like that I can keep all my reports in the electronic format	5.4	5.5	7	4	5.0	5	6	4	5.7	6	7	5
Perceived usefulness	5.9				5.5				6.2			
Using the system enhances the effectiveness of managing my health conditions	5.7	5.5	7	5	5.0	5	6	5	6.0	6	7	6
It explains me what my health status is	6.1	6	7	5	5.9	5.5	6	5	6.3	6.5	7	6
I can provide all the information about my test results to any doctor I visit	5.9	6	7	5	5.9	6	7	5	5.9	6	7	5

## Discussion

The paper describes a development of a patient facing clinical decision support system, which provides interpretation of the test results in the natural language.

### Notification ethics

We need to be very cautious when providing test results to the patients by e-mail or on a web portal. We should assume that the patients may not fully and properly comprehend the interpretation of the results. So, the capability to deliver results and their interpretation in a manner they are understood by a patient is essential for a motivation to refer to a health care professional, particularly when test results are abnormal. Our decision support system only interprets and sends test results that do not need a humane communication according to the standards of the laboratory service. Test results that can be communicated only in person include positive HIV test, all kinds of hepatitis and all kind of positive cancer tests.

The decision support system never intended to be prescriptive and communicate a single possible clinical decision to a patient. To follow this approach, we have implemented the reports in a way that they are descriptive and informative rather than prescriptive.

### Correctness

Rules definition process shall be controlled and always reviewed. The evaluation showed that the correctness of the generated reports is high. Seven mistakes out of 1000 analyzed reports were caused by a human factor. The mistakes that were detected by the experts during the assessment were caused by the inaccuracies of the experts when modelling inference rules. This led to a change of rules’ definition procedure, where we apply 4 eyes principle [34]. Now, when each rule goes to production only after a review and acceptance of a second expert.

### Use acceptance

One of the measures of feasibility was the percent of patients who agreed to take part in the testing of the decision support tool. The rate of 86% of patients who agreed to take part in the study shows high interest and motivation, which is supported by quantitative measurements that were done within the study.

The user acceptance of the system was evaluated after 2 months of operation. Acceptance scores were high, all of them above 5.7 out of 7. Among elder users (60+) the results were a little worse in comparison to the younger users. The maximum difference was 0.7 (10%) for the PU. Elder people felt less motivated about storing their medical data in electronic format (5.0 versus 5.7 for the younger participants). Maximum rates were similar for each statement and age group, and a high median value also indicates a positive attitude to the system. Minimum rates of 4 show an encouragement towards the system, as all the rates were in the positive part of the scale. This is true for all age groups. This indicates high acceptance of the solution and the way the notifications are being delivered.

Partial correlations calculated using scales derived for these dimensions suggested that Ease of Use and Usefulness impact one another in a way that enhancements in Ease of Use increase the scores of Usefulness and the other way round. Whereas both Ease of Use and Usefulness steer Satisfaction with Usefulness having comparatively less significant influence. Users are more flexible in their Usefulness scores when they have only reduced experience with a system.

Unfortunately, we could not compare them to the similar studies, as we did not find a patient-oriented decision support system, for which a user acceptance was evaluated. However, we tried to compare the results with similar systems that were not patient oriented.

Acceptance scores were relatively high compared to the results of evaluation results of previous studies [35–38] management smartphone app. This can be explained by the fact that most of the study participants had average or above average IT skills. This is in a contrast with the previous studies on the patients’ acceptance of decision support tools and can be explained by the increased computer literacy and changing IT habits. Our results mean that the health care providers and EHR developers can move in the direction of electronic notifications of the patients. This will facilitate communication and decrease its costs.

### Implications

The results of our study support other literature suggesting that patients want timely and detailed information and they want to be notified of all laboratory test results, even if they are normal [39, 40]. However, our results contradict the previous ones in regards to the patients preferring phone calls and sealed letters to the web based notification methods [40].

The findings of this research have valuable inferences for the design and implementation of patient notification systems. We found that patients in general find the detailed notifications useful, are motivated to use them and don’t face significant difficulties to adopt such solutions. It is very important when designing and implementing patients’ notification systems to make them valid, easy and simple to use. To achieve this, we advise that a pilot application of the decision support system is tested by the experts for verification and validation of the rules and potential users for the user acceptance, so that corrections can be made during the implementation phase to increase the system’s reliability and acceptance.

The results of our study suggest that there is a major impact of patients’ habits on the test results notification utilization. In addition to the unswerving and natural effect of habit on IT use, a habit also functions as a stowed purpose trail to affect behavior. Promotion of electronic test results notifications still demands major communication effort to strengthen both the stowed intention and its relation to behavior.

### Legal

It is important to mention that in Russia laboratory ervices are legally obliged to provide results of laboratory tests to patients. Providing not only the results but the explanations of their meaning will enhance the notifications and make them more valuable for patients.

### Limitations of the study and future work

#### Tool’s impact on patients’ decision-making

We did not thoroughly gather data on participants’ decision to follow up or not laboratory tests especially for the tests with abnormal results. This will become a major part of our next study where we will investigate how the patients decide to follow up or not laboratory tests. A systematic review by Callen et al. found that, across 19 published studies, 6.8–62% of lab tests were not followed up on [41, 42]. We think that this rate will increase for the patients that receive detailed information about the test results and their possible implications.

#### Evolving the decision support system

We are evolving the decision support system every day by adding new inference rules and optimizing its architecture. The next steps would be to add a possibility of working with fuzzy rules [43] to make the inference more flexible. Also, we are redesigning a data storage architecture to move from relational data base to a graph data base, that in our mind is more suitable for modeling knowledge and inference rules.

Mobile application for the patients is also under development now. This can potentially involve younger users to the system.

## Conclusions

The findings of the research provide us with a better understanding of how patients experience detailed notification of laboratory tests without health care professional participating in the process. Detailed notification of laboratory service patients with the elements of decision support is significant for laboratory data management, and for patients’ empowerment and safety. We suppose that patients empowered in such way can play a significant role in the process of delivering test results to the physicians, which positively affects the efficiently of a diagnostics and treatment process.

## Additional files

Additional file 1: Questionnaire. A Questionnaire to study the acceptance of the sytem by patients. (DOCX 63 kb)

Additional file 2: Input data for a decision support. Blood Sugar Test Results as an input for decision support. (DOCX 77 kb)

## Abbreviations

BI: Behavioral intention to use
EHR: Electronic health record
ICD: International Statistical Classification of Diseases and Related Health Problems
IM: Intrinsic motivation,
IT: Information technology
LIS: Laboratory information system
PC: Personal computer
PEOU: Perceived ease-of-use
PU: Perceived usefulness
