# Supplementary material for: Patient facing decision support system for interpretation of laboratory test results
Source: BMC Med Inform Decis Mak. 2018 Jul 20;18:68. doi: 10.1186/s12911-018-0648-0 (PMC6053711; doi:10.1186/s12911-018-0648-0)
Supplement: Supplementary file 1 — Questionnaire. A Questionnaire to study the acceptance of the sytem by patients. (DOCX 63 kb) [file 12911_2018_648_MOESM1_ESM.docx]

Additional file 1. Questionnaire

Dear Name SURNAME, You have been using the Helix laboratory test management site for quite a while now having more then 5 reports there. Please read carefully the statements bellow and rate them from 1 (not at all) to 7 (very much).

| Statement | Your rate (from 1 to 7) |
| --- | --- |
| I intend to use the tool to understand my test results |  |
| I feel like I will use it in the future |  |
| I find the system to be useful for me |  |
| The system helps me to make more informed decisions |  |
| The system is reliable and I trust it |  |
| The reports are clear and understandable |  |
| It is easy to access the reports online |  |
| I like that I can keep all my reports in the electronic format |  |
| Using the system enhances the effectiveness of managing my health conditions |  |
| The system explains me what my health status is |  |
| I can provide all the information about my test results to any doctor I visit |  |
