# Supplementary material for: Patient facing decision support system for interpretation of laboratory test results
Source: BMC Med Inform Decis Mak. 2018 Jul 20;18:68. doi: 10.1186/s12911-018-0648-0 (PMC6053711; doi:10.1186/s12911-018-0648-0)
Supplement: Supplementary file 2 — Input data for a decision support. Blood Sugar Test Results as an input for decision support. (DOCX 77 kb) [file 12911_2018_648_MOESM2_ESM.docx]

# Additional file 2. Input data for a decision support Blood Sugar Test

## Input data

{

"resourceType": "Bundle",

"entry": [{

"resource": {

"resourceType": "Patient",

"id": "cdbb5f61-6647-4e3b-9ddd-6fee30f68d6c",

"active": true,

"name": [{

"family": ["-"],

"given": ["-",

"-"]

}],

"gender": "female",

"birthDate": "2012-01-01"

}

},

{

"resource": {

"resourceType": "Observation",

"id": "urn:uuid:7b56e222-a90a-42c8-8d6e-669e087c5f2b",

"status": "final",

"code": {

"coding": [{

"system": "http://api.medlinx.online/terminology/helix-manufacture-nomenclature",

"code": "H580",

"display": "

HbA1C_COBAS:Concentration"

}],

"text": " HbA1C _COBAS:Concentration of HbA1C "

},

"subject": {

"reference": "Patient/cdbb5f61-6647-4e3b-9ddd-6fee30f68d6c"

},

"issued": "2017-12-13T00:29:53+03:00",

"valueQuantity": {

"value": 0.27

},

"interpretation": {

"coding": [{

"system": "http://hl7.org/fhir/v2/0078",

"code": "N"

}]

}

}

},

{

"resource": {

"resourceType": "Observation",

"id": "urn:uuid:2d06e443-6ce9-4446-9a14-246cfbfc21b5",

"status": "final",

"code": {

"coding": [{

"system": "http://loinc.org",

**"code": "4548-4",**

"display": " HbA1C_COBAS:Proportion of HbA1C, %"

}],

"text": " HbA1C_COBAS:Proportion, %"

},

"subject": {

"reference": "Patient/cdbb5f61-6647-4e3b-9ddd-6fee30f68d6c"

},

"issued": "2017-12-13T00:29:53+03:00",

"valueQuantity": {

"value": 4.6

},

"interpretation": {

"coding": [{

"system": "http://hl7.org/fhir/v2/0078",

"code": "L"

}]

},

"referenceRange": [{

"low": {

"value": 4.8

},

"high": {

"value": 6.0

}

}]

}

},

{

"resource": {

"resourceType": "Observation",

"id": "urn:uuid:013a08da-f25f-43d6-b102-79d3e43711db",

"status": "final",

"code": {

"coding": [{

"system": "http://api.medlinx.online/terminology/helix-manufacture-nomenclature",

"code": "H581",

"display": " HbA1C_COBAS:Concentration of Hb"

}],

"text": " HbA1C_COBAS:Concentration of Hb"

},

"subject": {

"reference": "Patient/cdbb5f61-6647-4e3b-9ddd-6fee30f68d6c"

},

"issued": "2017-12-13T00:29:53+03:00",

"valueQuantity": {

"value": 10.13

},

"interpretation": {

"coding": [{

"system": "http://hl7.org/fhir/v2/0078",

"code": "N"

}]

}

}

},

{

"resource": {

"resourceType": "Observation",

"id": "urn:uuid:02844771-581c-4674-ade3-88de75619ddd",

"status": "final",

"code": {

"coding": [{

"system": "http://loinc.org",

"code": "14749-6",

"display": "Glucose in Plasma_COBAS:Concentration"

}],

"text": " Glucose in Plasma_COBAS:Concentration "

},

"subject": {

"reference": "Patient/cdbb5f61-6647-4e3b-9ddd-6fee30f68d6c"

},

"issued": "2017-12-12T22:49:13+03:00",

"valueQuantity": {

"value": 4.6

},

"interpretation": {

"coding": [{

"system": "http://hl7.org/fhir/v2/0078",

"code": "N"

}]

},

"referenceRange": [{

"low": {

"value": 3.3

},

"high": {

"value": 5.6

}

}]

}

},

{

"resource": {

"resourceType": "Observation",

"id": "urn:uuid:8a917446-ea47-4c76-970d-77bc1a2548f9",

"status": "final",

"code": {

"coding": [{

"system": "http://loinc.org",

"code": "1986-9",

"display": "C-Peptide_COBAS:Concentration"

}],

"text": " C-Peptide_COBAS:Concentration"

},

"subject": {

"reference": "Patient/cdbb5f61-6647-4e3b-9ddd-6fee30f68d6c"

},

"issued": "2017-12-12T20:48:37+03:00",

"valueQuantity": {

"value": 1.15

},

"interpretation": {

"coding": [{

"system": "http://hl7.org/fhir/v2/0078",

"code": "N"

}]

},

"referenceRange": [{

"low": {

"value": 1.1

},

"high": {

"value": 4.4

}

}]

}

},

{

"resource": {

"resourceType": "DiagnosticReport",

"code": {

"coding": [{

"system": "http://api.medlinx.online/terminology/nomenclature",

"code": "41.010"

}]

},

"result": [{

"reference": "urn:uuid:7b56e222-a90a-42c8-8d6e-669e087c5f2b"

},

{

"reference": "urn:uuid:2d06e443-6ce9-4446-9a14-246cfbfc21b5"

},

{

"reference": "urn:uuid:013a08da-f25f-43d6-b102-79d3e43711db"

},

{

"reference": "urn:uuid:02844771-581c-4674-ade3-88de75619ddd"

},

{

"reference": "urn:uuid:8a917446-ea47-4c76-970d-77bc1a2548f9"

}]

}

}]

}

## Inference sequence

[{

**"Id": 4785,**

"Type": 2,

"LoincId": null,

"Description": null,

"CalculatedValue": true,

"InversionFlag": false,

"OperationType": "AND",

"GroupName": null,

"Severity": 0,

"ValueType": 0,

"LiteralCurrentValue": null,

"NumericCurrentValue": 0.0,

"NumericValue": 0.0,

"ReferenceHiValue": 0.0,

"ReferenceLowValue": 0.0,

"ReferenceLiteral": null,

"NumericRangeLowValue": 0.0,

"NumericRangeHiValue": 0.0,

"LiteralValue": null,

"Operation": 0,

"Children": [{

**"Id": 4695,**

"Type": 2,

"LoincId": null,

"Description": null,

"CalculatedValue": true,

"InversionFlag": false,

**"OperationType": "AND",**

"GroupName": null,

"Severity": 0,

"ValueType": 0,

"LiteralCurrentValue": null,

"NumericCurrentValue": 0.0,

"NumericValue": 0.0,

"ReferenceHiValue": 0.0,

"ReferenceLowValue": 0.0,

"ReferenceLiteral": null,

"NumericRangeLowValue": 0.0,

"NumericRangeHiValue": 0.0,

"LiteralValue": null,

"Operation": 0,

"Children": [{

**"Id": 4698,**

"Type": 1,

"LoincId": "1986-9",

"Description": null,

"CalculatedValue": true,

"InversionFlag": false,

**"OperationType": null,**

"GroupName": null,

"Severity": 0,

"ValueType": 1,

"LiteralCurrentValue": null,

"NumericCurrentValue": 1.15,

"NumericValue": 0.0,

"ReferenceHiValue": 4.4,

"ReferenceLowValue": 1.1,

"ReferenceLiteral": null,

"NumericRangeLowValue": 0.0,

"NumericRangeHiValue": 0.0,

"LiteralValue": null,

"Operation": 9,

"Children": null,

"Artefacts": null,

"Definitions": null

},

{

**"Id": 4697,**

"Type": 1,

"LoincId": "4548-4",

"Description": null,

"CalculatedValue": true,

"InversionFlag": false,

**"OperationType": null,**

"GroupName": null,

"Severity": 0,

"ValueType": 1,

"LiteralCurrentValue": null,

"NumericCurrentValue": 4.6,

"NumericValue": 0.0,

"ReferenceHiValue": 5.9,

"ReferenceLowValue": 4.8,

"ReferenceLiteral": null,

"NumericRangeLowValue": 0.0,

"NumericRangeHiValue": 0.0,

"LiteralValue": null,

"Operation": 11,

"Children": null,

"Artefacts": null,

"Definitions": null

},

{

**"Id": 4696,**

"Type": 1,

"LoincId": "14749-6",

"Description": null,

"CalculatedValue": true,

"InversionFlag": false,

**"OperationType": null,**

"GroupName": null,

"Severity": 0,

"ValueType": 1,

"LiteralCurrentValue": null,

"NumericCurrentValue": 4.6,

"NumericValue": 0.0,

"ReferenceHiValue": 5.6,

"ReferenceLowValue": 3.3,

"ReferenceLiteral": null,

"NumericRangeLowValue": 0.0,

"NumericRangeHiValue": 0.0,

"LiteralValue": null,

"Operation": 9,

"Children": null,

"Artefacts": null,

"Definitions": null

},

{

**"Id": 4807,**

"Type": 2,

"LoincId": null,

"Description": null,

"CalculatedValue": true,

"InversionFlag": false,

**"OperationType": "OR",**

"GroupName": null,

"Severity": 0,

"ValueType": 0,

"LiteralCurrentValue": null,

"NumericCurrentValue": 0.0,

"NumericValue": 0.0,

"ReferenceHiValue": 0.0,

"ReferenceLowValue": 0.0,

"ReferenceLiteral": null,

"NumericRangeLowValue": 0.0,

"NumericRangeHiValue": 0.0,

"LiteralValue": null,

"Operation": 0,

"Children": [{

**"Id": 4803,**

"Type": 2,

"LoincId": null,

"Description": null,

"CalculatedValue": true,

"InversionFlag": false,

**"OperationType": "AND",**

"GroupName": null,

"Severity": 0,

"ValueType": 0,

"LiteralCurrentValue": null,

"NumericCurrentValue": 0.0,

"NumericValue": 0.0,

"ReferenceHiValue": 0.0,

"ReferenceLowValue": 0.0,

"ReferenceLiteral": null,

"NumericRangeLowValue": 0.0,

"NumericRangeHiValue": 0.0,

"LiteralValue": null,

"Operation": 0,

"Children": [{

**"Id": 4806,**

"Type": 1,

"LoincId": "1986-9",

"Description": null,

"CalculatedValue": true,

"InversionFlag": false,

**"OperationType": null,**

"GroupName": null,

"Severity": 0,

"ValueType": 1,

"LiteralCurrentValue": null,

"NumericCurrentValue": 1.15,

"NumericValue": 0.0,

"ReferenceHiValue": 4.4,

"ReferenceLowValue": 1.1,

"ReferenceLiteral": null,

"NumericRangeLowValue": 0.0,

"NumericRangeHiValue": 0.0,

"LiteralValue": null,

"Operation": 8,

"Children": null,

"Artefacts": null,

"Definitions": null

},

{

**"Id": 4805,**

"Type": 1,

**"LoincId": "4548-4",**

"Description": null,

"CalculatedValue": true,

"InversionFlag": false,

"OperationType": null,

"GroupName": null,

"Severity": 0,

"ValueType": 1,

"LiteralCurrentValue": null,

"NumericCurrentValue": 4.6,

"NumericValue": 0.0,

"ReferenceHiValue": 5.9,

"ReferenceLowValue": 4.8,

"ReferenceLiteral": null,

"NumericRangeLowValue": 0.0,

"NumericRangeHiValue": 0.0,

"LiteralValue": null,

"Operation": 8,

"Children": null,

"Artefacts": null,

"Definitions": null

},

{

"**Id": 4804,**

"Type": 1,

**"LoincId": "14749-6",**

"Description": null,

"CalculatedValue": true,

"InversionFlag": false,

**"OperationType": null,**

"GroupName": null,

"Severity": 0,

"ValueType": 1,

"LiteralCurrentValue": null,

"NumericCurrentValue": 4.6,

"NumericValue": 0.0,

"ReferenceHiValue": 5.6,

"ReferenceLowValue": 3.3,

"ReferenceLiteral": null,

"NumericRangeLowValue": 0.0,

"NumericRangeHiValue": 0.0,

"LiteralValue": null,

"Operation": 8,

"Children": null,

"Artefacts": null,

"Definitions": null

}],

"Artefacts": null,

"Definitions": null

}],

"Artefacts": null,

"Definitions": null

}],

"Artefacts": null,

"Definitions": null

}],

"Artefacts": [{

**"Id": 4786,**

"Type": 4,

"LoincId": null,

"Description": "Text",

"CalculatedValue": false,

"InversionFlag": false,

"OperationType": null,

**"GroupName": "Hydrocarbon metabolism",**

"Severity": 0,

"ValueType": 0,

"LiteralCurrentValue": null,

"NumericCurrentValue": 0.0,

"NumericValue": 0.0,

"ReferenceHiValue": 0.0,

"ReferenceLowValue": 0.0,

"ReferenceLiteral": null,

"NumericRangeLowValue": 0.0,

"NumericRangeHiValue": 0.0,

"LiteralValue": null,

"Operation": 0,

"Children": null,

"Artefacts": null,

"Definitions": null

}],

"Definitions": null

}]

## Results of the inference

[{

"id": 4786,

"groupName": " Carbohydrate metabolism

",

"severity": 0,

"text": "

Measurement of a blood glucose level provides a valid information only for the time of a measurement. Glycated haemoglobin allows to measure an average co blood glucose level. С-peptide is a part of a proinsulin molecule. C-peptide level is a stable indicator of pancreas producing insulin than the quickly changing level of insulin itself \n.

The test showed a low level of Glycated haemoglobin along with a normal glucose and C-peptide level. This can indicate problems with hydrocarbon metabolism.

First of all it is necessary to exclude long term hypoglycaemic conditions (Hypoglicemia is condition when a blood glucose level is lower than reference values).

We recommend you visit an endocrinologist to decide on the future diagnostics and treatment

Preliminary Diagnosis: Hypoglycemia, unspecified. E16.2

}]
